# Stem Cell Differentiation and Therapeutic Use

**DOI:** 10.1155/2015/308128

**Published:** 2015-08-04

**Authors:** Matthew S. Alexander, Juan Carlos Casar, Norio Motohashi

**Affiliations:** ^1^Division of Genetics and Genomics, Boston Children's Hospital, Boston, MA 02115, USA; ^2^Department of Pediatrics and Genetics, Harvard Medical School, Boston, MA 02115, USA; ^3^The Stem Cell Program, Boston Children's Hospital, Boston, MA 02115, USA; ^4^Departamento de Neurología, Escuela de Medicina, Pontificia Universidad Catolica de Chile, Santiago 8330033, Chile; ^5^Department of Geriatric Medicine, Tokyo Metropolitan Institute of Gerontology, Tokyo 173-0015, Japan

Stem cell therapy is a promising approach to cure degenerative diseases, cancer, damaged tissues, or any disease for which there are very limited therapeutic options. Stem cell therapies could potentially improve the efficiency of the human body regenerative response following an injury or insult, in addition to being a source of powerful therapeutic compounds that hold the promise of the restoration of normal function of a given tissue. The abilities to identify, isolate, expand, and differentiate stem cells have been past barriers for developing therapies in patients and treatments using stem cells have long been viewed as potential or under development. The discovery of induced pluripotent stem cells (iPSC) has encouraged many researchers in the field of stem cell biology and organized-patient groups to advocate for increased development of stem cell-based therapies and in-depth scientific knowledge.

The field of regenerative medicine and the ability to harness and manipulate stem cells to treat human diseases have been opened by a combination of many technical advances [1]. A few, among these improved technologies, should be mentioned. Whole transcriptome analyses have allowed researchers to better understand the molecular signatures of different stem cell populations in human tissues and rodent models [2]. New methods in generating novel transgenic models have allowed researchers to perform elegant lineage tracing of stem cells as they proliferate and differentiate into specific somatic lineages [3–5]. Improvements in techniques to prospectively isolate stem cell populations, manipulate their genomes, and image/track them have also greatly improved in the past decade [6–8]. Advances in large scale genetic and drug library screening have also allowed an unprecedented insight into the molecular changes and factors necessary to convert pluripotent cells into progenitors of specific lineages [9].

Last year, clinical trials using retinal pigment epithelium derived from iPS cells were performed for patients who had severe age-related macular degradation in Japan [10, 11]. These trials evaluated the safety of iPS cells generated from the recipient's own cells and they have shown that the iPS cells did not evoke any immune reactions and did not produce tumors in mice and monkeys before conducting transplants. So far, no problems or health concerns have been reported, suggesting that iPS cells constitute an additional option for stem cell-based therapies. One remaining concern is whether the engrafted iPS cells will be working as part of the regenerating tissue and whether the transplanted cells will persist for a long time. Another exciting report this year was a US-based clinical trial from Robert Lanza's group at Advanced Cell Technology, demonstrating efficacy and tolerance of human embryonic stem (ES) cell-derived retinal pigment epithelium cells transplanted into patients with Stargardt's macular dystrophy [12]. This phase 1/2 clinical trial did not show any adverse effects and an improvement in visual function in a significant number of patients receiving the transplanted cells was observed. These findings have given patients with previously untreatable degenerative disorders hope for a potential regenerative stem-cell based treatment. The current state of development of stem cell-based therapies for different conditions is reviewed by J. F. Stoltz et al. and R. Compagna et al., in this issue. Additional potential for therapies may lie in the ability to harness the power of mesenchymal stem cells (MSCs). Mesenchymal stem cells are stem cells that developmentally originate from the embryonic mesoderm and are thought to be involved in the regeneration and tissue repair processes of several organs including the bone, vasculature, connective ligaments, and liver [13, 14]. It is this therapeutic potential of MSCs that makes them attractive for potential clinical therapies, as evident by studies of bone-marrow and tendon-derived mesenchymal stem cells of regenerative potential by M. K. Al-ani et al. in this issue. Additional work on the differentiation potential of various MSCs (such as that by D. J. Zhang et al. in this issue) into more somatic lineages highlights the progress made towards directed lineage conversion of specific stem cell populations.

Despite these promising initial studies, potential and unanticipated risks and complications or side effects might become apparent with progress of stem cell-based therapies. Continued research is fundamental for the evaluation and exclusion of potential risks before the clinical use of stem cells. It is difficult, however, to predict with certainty the risk of these therapies due to the number and complexity of the variables involved, such as type of stem or progenitor cells, their proliferation or differentiation capacity, the methods for their isolation and route of administration, the engraftment location, and others derived from the recipients' age or health condition. Rigorous preclinical experiments and safety trials are needed to address some of these issues whose design must take into account both observations from clinical experience and from animal studies including tumor formation and/or immune responses. Understanding the basic biology and differentiation potential of stem cells (as in the study of the SDF-1/CXCR4 axis as a regulator of MSCs to repair liver injury by N.-B. Hao et al. in this issue) might be an important clue to solve some of these downstream problems. It is well known that stem cells not only work as a part of tissue regeneration, but also secrete factors for maintenance and well-being of tissue homeostasis, for example, by promoting vasculature, suppressing inflammation, or accelerating cell growth. Interesting examples of these mechanisms are described in the studies by A. L. Strong et al. and D. J. Zhang et al. included in this issue.

A remaining significant hurdle for scientists and clinicians is the growing number of unregulated stem cell clinics [15]. False promises of miracle cures using stem cells have led to the burgeoning industry of stem cell tourism, where patients travel outside of countries with strong regulation of stem cell uses to countries where stem cell regulations are more lax [16]. As we move forward in advancing rational therapies for treating patients, it is important for the field to ensure patient safety and openness of sharing all clinical trial results, both positive and negative, with the public.

In this special issue, we present a collection of studies and reviews that highlight a broad variety of topics related to stem cell differentiation and potential therapeutic use. We hope that the readers will appreciate the amount of progress in addition to the challenges that have been made in the past decade towards understanding and characterizing stem cells from embryonic to adult stem cells. As is evident by the investment made by several governmental and private agencies, novel stem cell therapies are well on their way to becoming a reality for patients dealing with debilitating diseases.

*Matthew**  **S.**  **Alexander* *Matthew**  **S.**  **Alexander* *Juan**  **Carlos**  **Casar* *Juan**  **Carlos**  **Casar* *Norio**  **Motohashi* *Norio**  **Motohashi*

## References

[1] Fox I. J., Daley G. Q., Goldman S. A., Huard J., Kamp T. J., Trucco M. (2014). Use of differentiated pluripotent stem cells in replacement therapy for treating disease. *Science*.

[2] Wang Y., Navin N. E. (2015). Advances and applications of single-cell sequencing technologies. *Molecular Cell*.

[3] Branda C. S., Dymecki S. M. (2004). Talking about a revolution. *Developmental Cell*.

[4] Grompe M. (2012). Tissue stem cells: new tools and functional diversity. *Cell Stem Cell*.

[5] Hoppe P. S., Coutu D. L., Schroeder T. (2014). Single-cell technologies sharpen up mammalian stem cell research. *Nature Cell Biology*.

[6] Nguyen P. K., Riegler J., Wu J. C. (2014). Stem cell imaging: from bench to bedside. *Cell Stem Cell*.

[7] Healy K. E., McDevitt T. C., Murphy W. L., Nerem R. M. (2013). Engineering the emergence of stem cell therapeutics. *Science Translational Medicine*.

[8] Barker N., Bartfeld S., Clevers H. (2010). Tissue-resident adult stem cell populations of rapidly self-renewing organs. *Cell Stem Cell*.

[9] Zhang Y., Li W., Laurent T., Ding S. (2012). Small molecules, big roles—the chemical manipulation of stem cell fate and somatic cell reprogramming. *Journal of Cell Science*.

[10] Song P., Inagaki Y., Sugawara Y., Kokudo N. (2013). Perspectives on human clinical trials of therapies using iPS cells in Japan: reaching the forefront of stem-cell therapies. *BioScience Trends*.

[11] Inoue H., Nagata N., Kurokawa H., Yamanaka S. (2014). IPS cells: a game changer for future medicine. *The EMBO Journal*.

[12] Schwartz S. D., Regillo C. D., Lam B. L. (2015). Human embryonic stem cell-derived retinal pigment epithelium in patients with age-related macular degeneration and Stargardt's macular dystrophy: follow-up of two open-label phase 1/2 studies. *The Lancet*.

[13] Caplan A. I., Correa D. (2011). The MSC: an injury drugstore. *Cell Stem Cell*.

[14] Kfoury Y., Scadden D. T. (2015). Mesenchymal cell contributions to the stem cell niche. *Cell Stem Cell*.

[15] MacReady N. (2009). The murky ethics of stem-cell tourism. *The Lancet Oncology*.

[16] Crooks V. A., Snyder J. (2010). Regulating medical tourism. *The Lancet*.

